# A case of prolonged COVID‐19 treated with tixagevimab/cilgavimab

**DOI:** 10.1002/rcr2.1099

**Published:** 2023-02-09

**Authors:** Takahiro Shimizu, Hiroki Shirasaki, Kazuhiro Okafuji, Aiko Sawazaki, Tasuku Iwabuchi, Ryo Matubayashi

**Affiliations:** ^1^ Department of Internal Medicine Fukui Prefecture Saiseikai Hospital Fukui‐shi Japan

**Keywords:** COVID‐19, follicular lympoma, Obinutuzumab, SARS‐Cov‐2, tixagevimab/cilgavimab

## Abstract

A 54‐year‐old woman presented to our hospital with a fever and cough. The patient had a medical history of follicular lymphoma treated with obinutuzumab. She was infected with an omicron variant of coronavirus disease 2019 and developed viral pneumonia. Antibiotics, molnupiravir, sotrovimab, and prednisolone were administered but were ineffective. The patient's symptoms and pneumonia persisted. She could not produce antibodies against severe acute respiratory syndrome coronavirus 2 (SARS‐CoV‐2) because she was administered obinutuzumab. Finally, when we administered 300 mg of tixagevimab and cilgavimab, pneumonia immediately improved. However, the infection was prolonged for more than 4 months. Patients treated with anti‐CD20 monoclonal antibodies may have a prolonged SARS‐CoV‐2 infection. In such cases, tixagevimab/cilgavimab may be effective.

## Graphical abstract

A 54‐year‐old patient with COVID‐19 had a medical history of follicular lymphoma treated with obinutuzumab, an anti‐CD20 monoclonal antibody. This prevented the production of antibodies against severe acute respiratory syndrome coronavirus 2 (SARS‐CoV‐2), thus resulting in persistent pneumonia. Administration of 300 mg of tixagevimab and cilgavimab markedly improved the patient's general condition, and led to remittance of pneumonia.

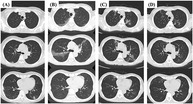

## INTRODUCTION

Severe acute respiratory syndrome coronavirus 2 (SARS‐CoV‐2) is an endemic virus that affects people worldwide. Patients with coronavirus disease 2019 (COVID‐19) treated with anti‐CD20 antibodies have worse outcomes and prolonged symptoms.^1^ However, no evidence‐based treatment is available for these patients. Herein, we report a case of prolonged COVID‐19 that was treated with tixagevimab/cilgavimab.

## CASE REPORT

**FIGURE 1:**
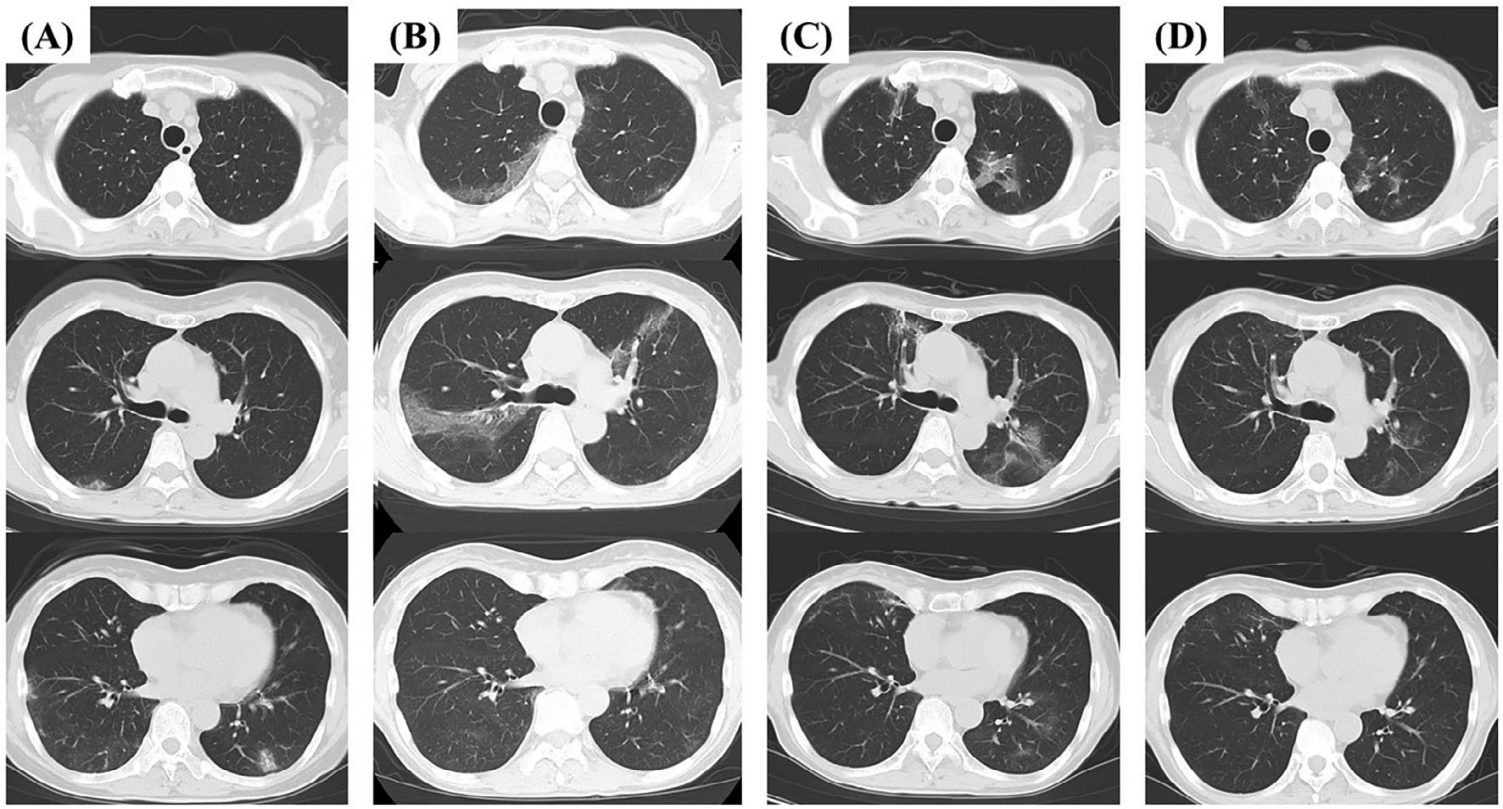
(A) Subpleural ground‐glass opacities were seen in both lungs. (B) Ground‐glass opacities were migrated and worse after antibiotics and prednisolone administration. (C) The shadows remained after sotrovimab administration. (D) These findings were improved after 1 week after the administration of tixagevimab/cilgavimab

A 54‐year‐old woman presented to our hospital with a fever and cough. The patient had a medical history of follicular lymphoma treated with obinutuzumab, an anti‐CD20 monoclonal antibody, and was in complete remission. She did not receive the SARS‐CoV‐2 vaccination. She was diagnosed with COVID‐19 using a nasopharyngeal SARS‐CoV‐2 polymerase chain reaction (PCR) test 1 month earlier. At the time, SARS‐CoV‐2 omicron variants were prevalent in Japan, and she was probably infected. The patient was treated with molnupiravir. Her symptoms became mild but flared shortly thereafter. Upon presentation, the patient's body temperature was 38.6°C and percutaneous oxygen saturation was 92% on room air. Chest computed tomography (CT) revealed subpleural ground‐glass opacities (Figure 1A). The patient was hospitalized for community‐acquired pneumonia. Ceftriaxone and levofloxacin were administered, but her fever persisted. Prednisolone was also administered, considering organizing pneumonia after infection. The patient's symptoms initially improved but soon flared. CT showed ground‐glass opacities that had migrated and worsened from the previous CT scan (Figure 1B). Nasopharyngeal SARS‐CoV‐2 PCR and antigen swab tests were mainly positive. Hypogammaglobulinemia and the absence of SARS‐CoV‐2 antibodies were determined. The final diagnosis was prolonged COVID‐19 due to a lack of SARS‐CoV‐2 antibodies after obinutuzumab treatment. Sotrovimab—a SARS‐CoV‐2 neutralizing monoclonal antibody—was administered, but pneumonia persisted (Figure 1C). This might have been because sotrovimab may have a reduced neutralizing activity against SARS‐CoV‐2 omicron variants.^2^ Finally, when we administered 300 mg of tixagevimab and cilgavimab, the patient's general condition improved markedly, and pneumonia remitted 1 week after the administration of these agents(Figure 1D). However, the infection was prolonged for more than 4 months from the onset of COVID‐19.

## DISCUSSION

Herein, we report a case of prolonged COVID‐19 after obinutuzumab treatment. The patient had the infection for over 4 months, which only improved with tixagevimab/cilgavimab treatment.

Obinutuzumab is an anti‐CD20 monoclonal antibody that induces humoral immunodeficiency by depleting B‐lymphocytes. COVID‐19 recurrence, proliferation, and severity have been reported in patients treated with anti‐CD20 monoclonal antibodies.^1^ After infection with SARS‐CoV‐2, the seroconversion rate is only 59% in patients previously treated with anti‐CD‐20 antibodies.^3^ In patients with hematologic malignancies who were infected with omicron variants, treatment with obinutuzumab resulted in worse clinical outcomes than treatment with rituximab.^1^

To our knowledge, this is the first case of administering tixagevimab/cilgavimab for prolonged COVID‐19 in a patient previously treated with anti‐CD‐20 antibodies. SARS‐CoV‐2 neutralizing monoclonal antibodies are effective in treating COVID‐19 cases that have a high risk of progressing to severe COVID‐19. However, casilivimab/imdevimab and sotrovimab have a significantly reduced neutralizing activity against omicron variants.^2^ Tixagevimab/cilgavimab binds to distinct, non‐overlapping epitopes on the spike protein receptor‐binding domain to neutralize SARS‐CoV‐2. These drugs also have a reduced neutralizing activity against omicron variants. Reportedly, administering 150 mg of these drugs is not effective against omicron variants in vitro, whereas 300 mg is effective.^4^ Tixagevimab/cilgavimab can be an effective treatment against omicron variants depending on the dose. The omicron BA5 variants were previously prevalent in patients with COVID‐19; however, the BQ.1.1 and XBB variants are increasingly prevalent. These subvariants have additional mutations in the receptor‐binding domain of the spike protein. An in vitro study showed that monoclonal antibodies, including tixagevimab/cilgavimab, are unable to neutralize the BQ.1.1 and XBB variants.^5^ When BQ.1.1 and XBB are prevalent, tixagevimab/cilgavimab may also be ineffective in the clinical setting.

Herein, we report a case of prolonged COVID‐19 that was treated with tixagevimab/cilgavimab. Tixagevimab/cilgavimab may be useful in the case of absence SARS‐CoV‐2 antibodies.

## AUTHOR CONTRIBUTIONS

Takahiro Shimizu was responsible for conceptualization and drafting the manuscript. Takahiro Shimizu, Hiroki Shirasaki, Kazuhiro Okafuji, Aiko Sawazaki, Tasuku Iwabuchi, Ryo Matubayashi analysed and interpreted the clinical and radiological data. All authors read and approved the final manuscript.

## FUNDING INFORMATION

No funding to declare.

## CONFLICT OF INTEREST STATEMENT

The authors declare no conflicts of interest.

## ETHICS STATEMENT

The authors declare that appropriate written informed consent was obtained for the publication of this manuscript and accompanying images.

## Data Availability

The data that support the findings of this study are available from the corresponding author upon reasonable request.

## References

[1] Shafat T , Grupel D , Porges T , Levi I , Yagel Y , Nesher L . Treatment with obinutuzumab leads to worse outcomes in haematological patients diagnosed with omicron variant COVID‐19. Br J Haematol. 2022;198(5):826–9. 35718461 10.1111/bjh.18315 PMC9350211

[2] Takashita E , Yamayoshi S , Simon V , van Bakel H , Sordillo EM , Pekosz A , et al. Efficacy of antibodies and antiviral drugs against omicron BA.2.12.1, BA.4, and BA.5 subvariants. N Engl J Med. 2022;387(5):468–70. 35857646 10.1056/NEJMc2207519 PMC9342381

[3] Thakkar A , Pradhan K , Jindal S , Cui Z , Rockwell B , Shah AP , et al. Patterns of seroconversion for SARS‐CoV‐2 IgG in patients with malignant disease and association with anticancer therapy. Nat Cancer. 2021;2(4):392–9. 34661163 10.1038/s43018-021-00191-y PMC8519533

[4] Stuver R , Shah GL , Korde NS , Roeker LE , Mato AR , Batlevi CL , et al. Activity of AZD7442 (tixagevimab‐cilgavimab) against omicron SARS‐CoV‐2 in patients with hematologic malignancies. Cancer Cell. 2022;40(6):590–1. 35598602 10.1016/j.ccell.2022.05.007 PMC9108069

[5] Imai M , Ito M , Kiso M , Yamayoshi S , Uraki R , Fukushi S , et al. Efficacy of antiviral agents against omicron subvariants BQ.1.1 and XBB. N Engl J Med. 2023;388(1):89–91. 36476720 10.1056/NEJMc2214302 PMC9749618

